# Alteration in global motor strategy following lateral ankle sprain

**DOI:** 10.1186/1471-2474-15-436

**Published:** 2014-12-16

**Authors:** Maude Bastien, Hélène Moffet, Laurent J Bouyer, Marc Perron, Luc J Hébert, Jean Leblond

**Affiliations:** Faculty of Medicine, Rehabilitation Department, Laval University, Quebec, QC Canada; Centre for Interdisciplinary Research and Social Integration (CIRRIS), Quebec Rehabilitation Institute, 525 boulevard Hamel, G1M 2S8 Quebec, QC Canada; National Defense of Canada- Canadian Forces Health, Quebec, QC Canada

**Keywords:** SEBT, Motor control, Musculoskeletal disorders, Ankle sprain

## Abstract

**Background:**

Lateral ankle sprain (LAS) has often been considered an injury leading to localized joint impairments affecting the musculoskeletal system. Persistent chronic ankle instability and bilateral alterations in motor control after a first ankle sprain episode suggest that the origin of relapses might be a maladaptive reorganization of central motor commands. The objectives of this study were (1) to compare the quality of motor control through motor strategy variables of two groups (with and without LAS) from a military population (n = 10/group), (2) to evaluate the contribution of the lower limbs and the trunk to global body strategy and (3) to identify which global variable best estimates performance on the Star Excursion Balance Test (SEBT) for each group, reaching direction, and lower limb.

**Methods:**

Personal and clinical characteristics of the participants of both groups were collected. Their functional ability was measured using questionnaires and they performed a series of functional tests including the SEBT. During this test, the maximal reach distance (MRD) and biomechanical data were collected to characterize whole body and segmental strategies using a 3D motion capture system.

**Results:**

At maximal lower limb reach, participants with LAS had a smaller variation in their vertical velocity in lowering-straightening and lowered the body centre of mass less for all injured limb conditions and some conditions with the uninjured lower limb. The global body centre of mass variables were significantly correlated to SEBT performance (MRD).

**Conclusion:**

Modifications in global motor strategies were found in participants with LAS as well as a decreased performance on the SEBT for the injured and uninjured lower limbs. These results support the hypothesis that following LAS, there may be a maladaptive reorganization of the central motor commands. Level of evidence: 3b.

**Electronic supplementary material:**

The online version of this article (doi:10.1186/1471-2474-15-436) contains supplementary material, which is available to authorized users.

## Background

Lateral ankle sprain (LAS) is one of the most commonly reported sports injuries among athletes and military personnel [1–5]. The military is the most affected population with a five to eight times greater prevalence than civilians [5–7]. Up to 33% of the civilians experience chronic ankle instability even in the absence of persistent ligament laxity around the ankle joint, as well as recurrence of LAS over the years [8]. Deficits in the limb contralateral to the injury such as postural control change in single-limb stance and altered transverse plane kinematic profiles in jump landing, have also been reported [9–16]. Taken together, these deficits suggest that motor control impairments may be at the origin of the relapses. It is therefore important to better understand the pathophysiology and types of motor control deficits during the early recovery phase after LAS to prevent recurrence and problems on the long term from which patients may not fully recovered such as chronic ankle instability [17, 18].

The persistence of alterations in motor control for the injured and uninjured limbs strongly supports the hypothesis of a reorganization of central motor commands. LAS causes swelling, pain and other peripheral damage. This leads to altered sensory inputs, which trigger a reorganization in sensorimotor processing leading to long-term central modification in movement planning and execution [19–21]. Such a sensorimotor processing deficit could explain the bilateral effects observed in participants with chronic ankle instability. Regarding potential neural control mechanisms that could be altered after LAS, it is known that individuals with chronic ankle instability have impaired sensorimotor function [11, 13, 22–26], and differences in segmental motor strategies during walking, running and jump landing [26–31].

In the present study, we were interested in looking at the changes in motor control strategies after LAS during a standardized motor task, as such changes give insight on the quality of motor control of the target population. To address this question, healthy participants and patients with LAS were tested in a challenging, yet standardized motor task that involves the coordination of multiple limb segments as well as good motor planning, i.e. that requires an optimal motor control [32–35].

The Star Excursion Balance Test (SEBT) was therefore selected. It has good metrological properties and has been frequently used to study motor control in athletes [9, 36–46]. The SEBT is defined by Gribble et al. [46] as “a series of single-limb squats using the nonstance limb to reach maximally to touch a point along 1 of 8 designated lines on the ground. The lines are arranged in a grid that extends from a center point and are 45° from one another. Each reaching direction offers different challenges and requires combinations of sagittal, frontal, and transverse movements” [46]. Maximal reach distance is the variable usually used to characterize performance and to identify alterations in motor control.

This variable reflects end-point control of the self-organization of movement. However, no study has used whole body kinematics to look at the global organization of movement contributing to the good performances observed on the SEBT. Such measures could provide additional information on SEBT performances and help identify where exactly, in the continuum of global motor strategies, the bilateral alterations of motor control take place. To date, only segmental strategies at the lower limb in stance have been described [47–49]. As motor control involves the ability to control the body centre of mass (CoM) over a base of support through appropriate coordination between segments [50–52], it seems important to also study its behaviour. The body CoM was chosen instead of body centre of pressure (CoP) since CoM takes into account all segmental strategies at the lower limbs, head and trunk and represents their summation in 3D coordinates. On the contrary, CoP commonly used in previous studies [11, 14, 53], is a biplanar variable with some limitation to represent 3D behaviors during a complex task. Therefore, in the present study, global body strategy was estimated by quantifying global body CoM 3D behaviour to appreciate the quality of motor control during the SEBT.

### Objectives

The objectives of this study were:

1. to compare the quality of motor control in two groups of military personnel (with and without LAS) on the SEBT, using the selected global and segmental body strategy variable;
2. to evaluate the contribution of the lower limbs and trunk to global body strategy for each group of participants and for each reaching direction; and
3. to identify which global variable used to quantify global body CoM behaviour best estimates performance on the SEBT for each group, reaching direction, and lower limb.

## Methods

### Participant selection

All volunteers participating in this study were selected from the Canadian Forces (CF) military population. The LAS group was composed of 10 men with a diagnosis of acute unilateral LAS [18]. They all received physiotherapy interventions within five days of injury and were discharged after a maximum of nine weeks. They were excluded if they had an ankle fracture (documented by X-ray), a third grade LAS or a high ankle sprain (tibiofibular sprain) [54–57], or if they reported a history of neuromuscular or neurodegenerative disease. The control group consisted of a comparable group of 10 men without prior history of LAS or symptoms from the lumbar spine and the lower limbs, or a history of neuromuscular and neurodegenerative disease. The present study was approved by the ethics committees of the Quebec Rehabilitation Institute and the CF Health Services Group. All participants read and signed an informed consent form.

### Study design

In this descriptive study, all participants were evaluated within a single session. Participants in the LAS group were selected in acute stage (within two days after injury) and were evaluated between eight and ten weeks after their injury. Personal and clinical characteristics of the participants were collected. Their functional ability was measured using two questionnaires: the Foot & Ankle Disability Index (FADI) [58–60] and the Lower Extremity Function Scale (LEFS) [61]. Thereafter, they took part in a series of complex motor tasks and tests, where the testing order was kept the same for all participants.

### SEBT procedure

**Figure 1:**
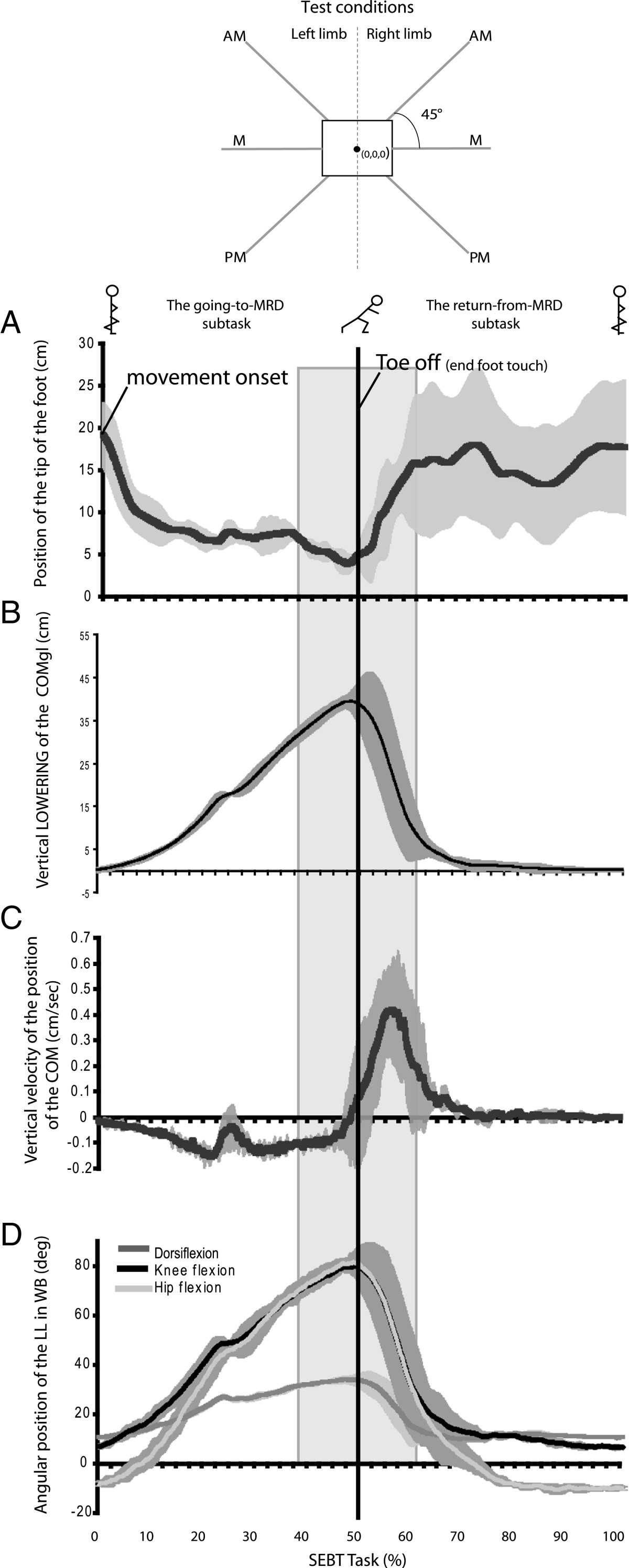
**Kinematics variables during the SEBT task, experimental setting and description of subtasks and events.** Illustration of the six conditions at the SEBT (3 reaching directions per limb; AM: anteromedial, M: medial, PM: posteromedial) and profiles (mean ± 1 standard deviation; n = 3 trials) of the vertical position of the reaching foot **(A)**, global body CoM (CoMgl) **(B** and **C)** and joint amplitude of motion of the stance limb **(D)** in a typical healthy subject in the PM reaching direction. The central grey rectangle, called the central phase around foot contact, represents the critical period used for the analyses. The lowest vertical position of the tip of the reaching foot was used to subdivide the task, which corresponds to the “foot contact” event (vertical line at 50% of the task). The first subtask is characterized by body lowering **(B** and **D)** and alignment of the reaching limb for foot contact **(A)**. In the second subtask which occurred after foot contact, a rapid straightening up of the entire body **(B** and **C)** is performed while returning from perturbation in single-limb stance. The transition between subtasks represents a highly challenging period for stability and motor control as a change in movement direction takes place at the perceived limits of stability. This transition period, called the central phase, was defined as -1 s to +1 s after foot contact. Further analysis about the quality of motor control was performed during this phase.

During the SEBT, the participant had to: (1) touch the floor as far as possible with the tip of the foot of the reaching limb in three different directions with respect to the stance limb (anteromedial [AM], medial [M] and posteromedial [PM] directions) and (2) return to unipedal stance after each reaching movement while maintaining balance on the stance limb with the hands resting on the iliac crests. The AM, M and PM directions were the ones proposed by Hertel et al. [62] because these directions were able to identify significant reach deficits associated with chronic ankle instability and provided complementary and non-redundant information. After a practice session [37, 63] (six trials/direction followed by a five-minute rest period), three successful trials, separated by a ten-second rest period, were recorded for each direction (Figure 1). Trials were rejected according to the criteria used in previous studies [62, 64, 65] (1) weight-bearing by the reaching limb, (2) displacement of the stance limb, or (3) loss of balance. As shown in Figure 1, the directions to be followed by the reaching limb during the tests were clearly marked on the floor using graduated tape. The order of test conditions (two legs and three directions) was determined as follows: first the leg order was randomly selected, then a test direction was randomly determined. All conditions for one leg were completed starting with the determined direction followed by the next anticlockwise direction (right leg) or clockwise direction (left leg). The same condition order was then used for the second leg.

### Data collection

During the SEBT, biomechanical data were collected to characterize both whole body and segmental strategies using a 3D motion capture system (Optotrak 3020; Northern Digital Inc., Waterloo, Ontario, Canada). Forty-five infrared markers placed in non-colinear triads were installed on the following body segments: lower limbs, head, and trunk. For uppers limbs, rings of three markers were placed around the wrists. Kinematic data was sampled at 50 Hz and then digitally filtered at 10Hz (low pass filter). Global body CoM (CoMgl) was estimated using the equations proposed by Winter [66, 67], and adjusted to consider the influence of the upper limb CoM^a^.

### Data analysis

All individual kinematic data analyses were based on a mean of three trials per test condition. A mean of two trials was exceptionally used in a very few cases (5 out of 120 conditions). The maximal reach distance (MRD) on the SEBT was calculated from the horizontal plane coordinates (vectorial distance) of the tip of the reaching foot (probed point) at the time it touched the floor.

#### Key variables used to characterize motor control

**Figure 2:**
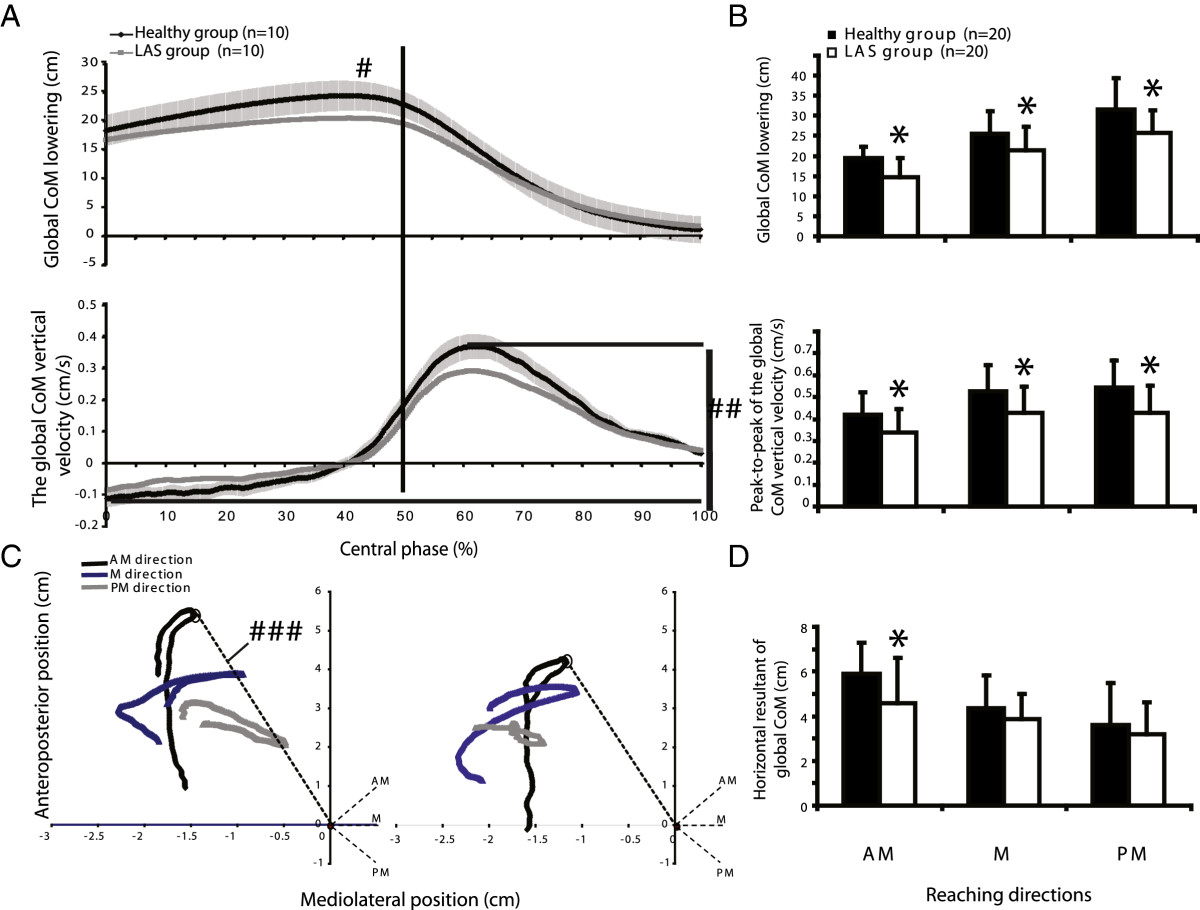
**Global body strategies along the vertical axis (A) and horizontal plane (C) in the central phase. A**: the profiles for global CoM lowering (#) and for the peak-to peak velocity of the global CoM (# #) are illustrated for one limb of both groups (mean ± 1 standard deviation for healthy group and mean for LAS group; n = 10 trials per group) during the medial reaching direction on the SEBT. **B**: mean values (+1 standard deviation) for global CoM lowering and peak-to peak of CoM vertical velocity of both groups. **C**: CoM displacement in the horizontal plane (# # #) during the different conditions (directions and limbs) in both groups. Horizontal resultant lines of global CoM position at foot contact (doted circles) were calculated and used for further statistical analyses. **D**: mean values (+1 standard deviation) for global CoM in the horizontal plane. Asterisks in figures B and D represent a significant difference between groups (MANOVA; *p* < 0.05; n = 20 limbs per group).

The SEBT task was divided into two main subtasks: the *going-to-* and the *return-from-MRD* (Figure 1A). The transition between these subtasks has been called the central phase and was defined as -1 s to +1 s after foot contact (Figure 1: central grey rectangle). Four variables describing the behaviour of the CoMgl, called global variables, have been chosen as key indicators of global body strategy during this task. The *maximal displacement of the CoMgl* vertically; *peak-to-peak CoMgl velocities* along the vertical axis were calculated during the central phase (Figure 2A); *distance between the centre of the foot and the mean position of the horizontal CoMgl* when the foot contacted the floor and the *horizontal excursion of the CoMgl* during the central phase were also calculated (Figure 2C).

In addition, the following variables representing the limb and trunk strategies during the task were quantified: maximal trunk lowering relative to the pelvis, maximal pelvis lowering, and magnitude of hip abduction of the reaching limb. Stance limb strategy was further analysed using the maximal peak of flexion of three joints (hip, knee and ankle) as well as their angular velocity in the sagittal plane (peak to peak value). Finally, for the reaching limb, angular velocity in the frontal plane at the hip was also computed.

#### Statistical analyses

**Figure 3:**
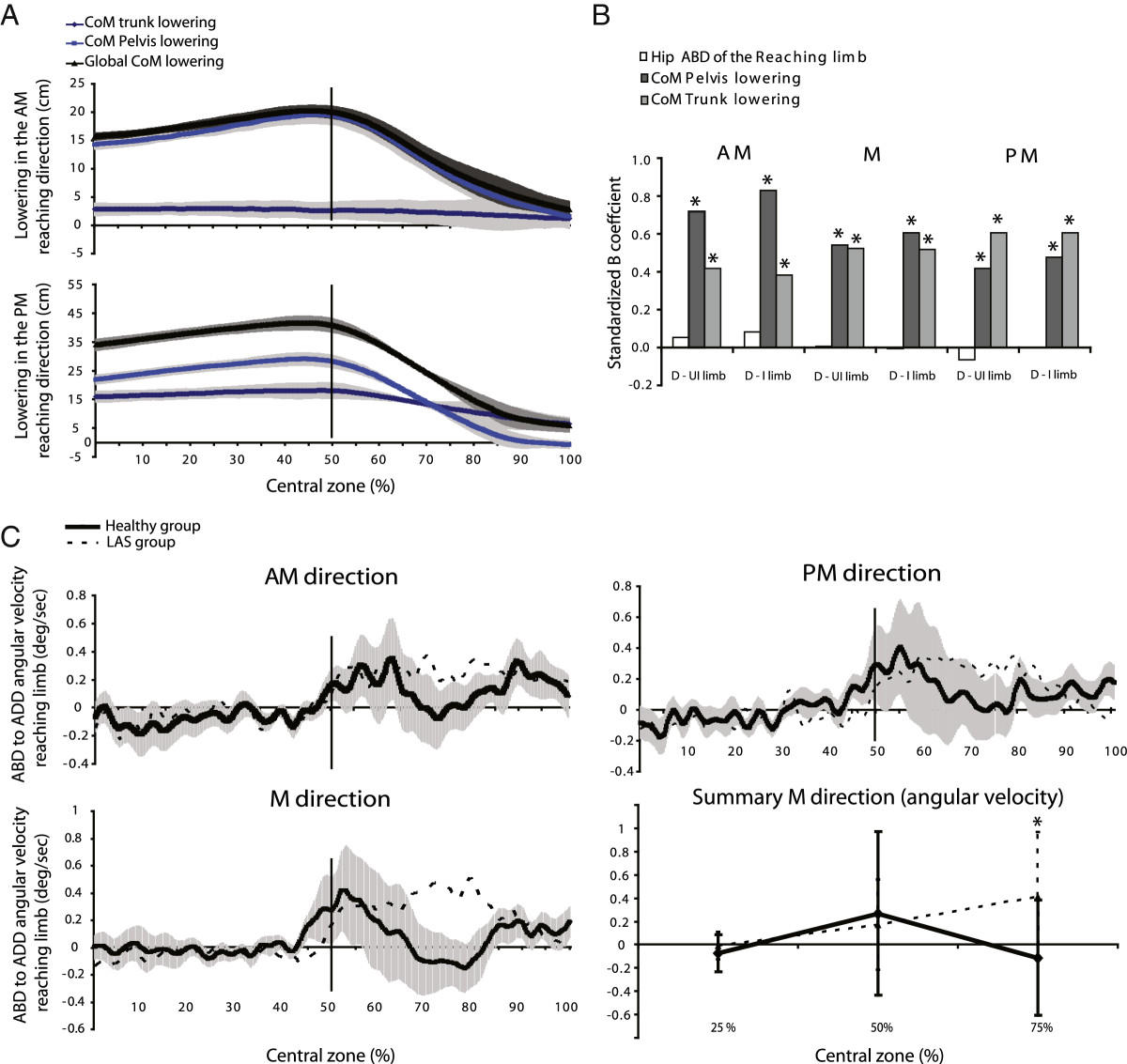
**Segmental motor strategy variables derived from global motor strategy variables. A**: Examples (mean profiles ±1 standard deviation) for a typical participant of different contributions of the pelvis and trunk lowering to the global CoM lowering between AM and PM directions in the central phase. **B**: Contribution of segmental strategy variables to the global CoM lowering represented by beta standardized coefficients (β) for each reaching direction and each lower limb. Each bar graph represents a combination of the dominant limb of the healthy group with either the uninjured limb (D-UI limb) or the injured limb (D-I limb) of the LAS group (n = 20). **C**: Hip ADD angular velocities of the reaching limb for the injured limb (dotted line) and the control group (black line and shaded area; mean ± 1 standard deviation) in each direction and a summary (mean velocity ±1 SD) at three different time points in the central phase for the medial direction. Asterisks represent a significant difference between groups (MANOVA; p < 0.05).

All statistical analyses (α = 0.05) were conducted using SPSS for Windows version 12.0. Parametric and descriptive analyses were conducted to meet the research objectives. Non-parametric tests (Mann–Whitney exact test) were used for the comparison of personal characteristics between groups. Descriptive statistics were calculated from the global strategy variables (Objective 1). Multivariate analyses were used to measure group and limb effects on global and segmental strategy variables. No results of univariate ANOVA were reported if the multivariate statistics were not found statistically significant (Objective 1). The horizontal excursion of the CoMgl was analysed using graphical representations (Objective 1). Mean difference (MD) and 95% confidence interval (CI) were calculated for statistically significant results. Regression analyses were carried out to determine which combination of two global variables along the vertical axis was providing the best estimate of SEBT performance for each test condition (using standardized Beta coefficients [β]) (Objective 3).As the majority of the LAS participants (70%) had injured their dominant limb, performance and strategy variables in all test conditions of the LAS participants were compared, for the second research objective, to those obtained from the dominant limb of the healthy group. A bar graph representing all regression coefficient combinations was used to compare each component’s contributions to global lowering for each reaching direction (see Figure 3, Objective 2). Peak-to-peak angular velocities in the stance limb were compared through multivariate analyses to measure the group and limb effects (Objective 1). The hip abduction velocity of the reaching limb was analysed at three times of measure (25, 50 and 75% of the central phase duration) to look for potential group effects for the injured and the uninjured limbs (repeated measured ANOVA) (Objective 1).

## Results

Participants in both healthy and LAS groups were similar in age (means ± 1SD: 26.1 ± 5.1 years and 26.3 ± 6.9 years, respectively), height, weight and lower limbs length. The LAS group had, however, a lower level of functional ability as measured by two questionnaires (FADI sport module: 79.4 ± 18.4 compared to 98.1 ± 2.6; LEFS: 73.3 ± 6.8 compared to 79.1 ± 1.4; *p* < 0.05).

### Quality of motor control and comparison of global body strategies between groups

**Figure 4:**
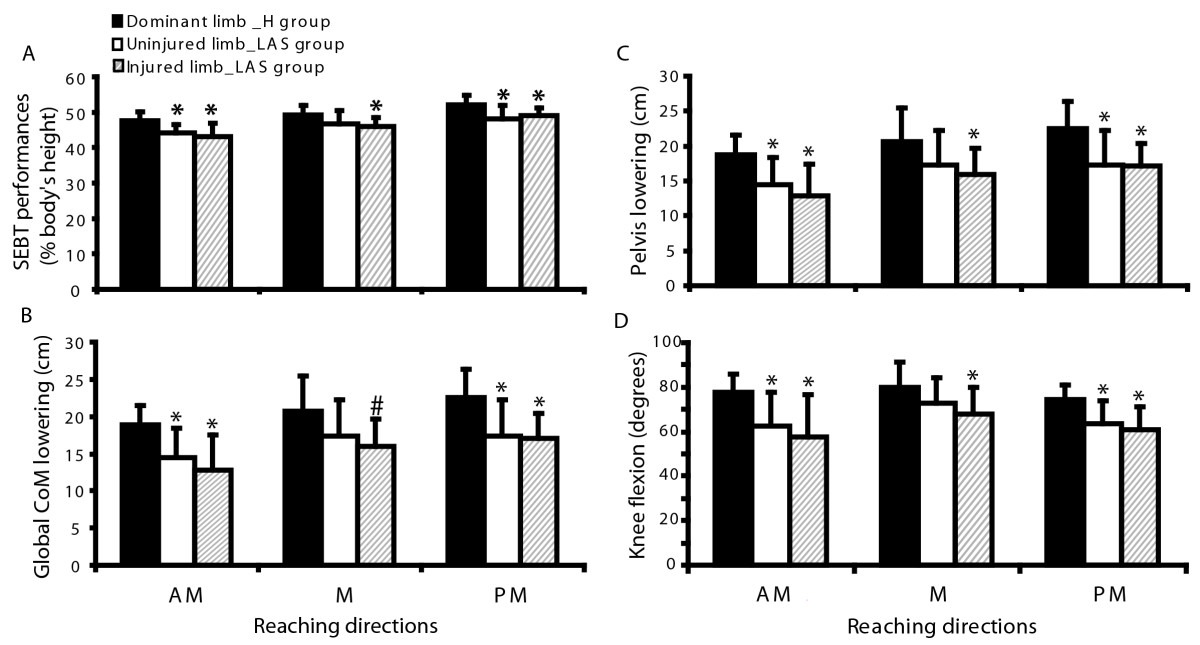
**Comparison of SEBT performance and motor strategies variables associated with CoMgl lowering between limbs (injured and uninjured) of the LAS group and the dominant limb of the healthy group for each direction.** Bar graphs represent the mean (n = 10 limbs per direction) and 1 standard deviation. Significant differences between groups are identified by asterisk (MANOVA test; *p <0.05; # *p* = 0.052). **A**: SEBT performance (maximal reach distance) expressed in percentage of the body height. **B**: Global motor strategy: Global CoM lowering, **C**: Segmental motor strategy: Pelvis lowering and **D**: Segmental motor strategy: Maximal knee flexion.

Around “foot contact,” the LAS group shows a smaller magnitude of CoMgl lowering (MD, 95% CI: 4.17 cm, (0.51 to 7.82) to 5.92 cm, (1.56 to 10.28), *p* < 0.05) and a smaller peak-to-peak CoMgl vertical velocities than the healthy group in all directions (0.08 cm/s, (0.01 to 0.15) to 0.12 cm/s, (0.04 to 0.20), *p* < 0.05) (Figure 2A). These results suggest that global body strategy along the vertical axis around foot contact is modified following LAS. There were significant differences between the injured and uninjured limb compared to the dominant limb of the healthy group for the CoMgl lowering variable in AM and in PM directions (MD, (95% CI): 4.57 cm, (0.85 to 8.28) to 7.18 cm (0.88 to 13.48), *p* value <0.05) (Figure 4B). The healthy group performed better at SEBT in all directions (Figure 4A), as measured by the MRD, except for the uninjured limb in the M direction (MD, (95% CI): 3.13% of height, (0.87 to 5.40) to 4.56% of height, (1.47 to 7.65), *p* value <0.05). They also significantly lowered their pelvis more and flexed the knee of the stance limb more than participants with LAS except for the uninjured limb in the M direction (see* = *p* <0.05; Figure 4C-D). The difference in maximal knee flexion between groups for the injured and dominant limb varied from 11.92° (95% CI: 0.98 to 22.85) (M direction) to 20.20° (95% CI: 5.72 to 34.69) (AM direction; Figure 4D). No difference across groups was found for other variables such as the maximal amplitude of ankle dorsiflexion (except in the AM direction, MD, (95% CI): 6.11°, (0.03 to 12.18)), hip flexion or relative trunk lowering.

A more detailed analysis of the peak-to-peak of CoMgl vertical velocity showed that only the injured limb was significantly different from the healthy group (ranges: healthy group 0.44 to 0.53 cm/s, LAS group 0.36 to 0.46 cm/s). Although no significant difference in peak-to-peak angular velocity at the stance limb joints was detected except for the ankle of the injured limb in PM direction, a tendency for larger values in the healthy group compared to the LAS was observed. The hip adduction velocity for the reaching limb of the LAS group was different from the healthy group in the central phase (Figure 3C). As shown in Figure 3, the LAS group brought back the reaching limb at a more constant velocity, as opposed to the healthy group, which used a bell-curve velocity profile before and after foot contact. No significant difference statistically supported this difference although this tendency except in the case of the injured limb in M direction (*p* = 0.04).

In the horizontal plane, during the central phase, CoMgl was progressively moving from a more centred position according to the stance foot to the perceived limit of stability, which is characterized by a U shape (Figure 2C). A descriptive analysis of the profiles of both groups highlights similarities in the general pattern of CoMgl displacement but also differences in the magnitude of the horizontal CoMgl resultant line. Indeed, for both groups, the projection of the CoMgl was located in the anterior and lateral section of the stance foot. This distance was significantly longer in the healthy group when compared to the LAS group (healthy group, 5.91 ± 1.38 cm; LAS group, 4.59 ± 2.1 cm; MD, (95% CI): 1.31 cm, (0.21 to 2.42), *p* < 0.05) in the AM reaching direction (Figure 2D). However, not shown in these figures were also significant differences between the injured and the uninjured limb compared to the dominant limb of the healthy group for the same direction (*p* value <0.05). No differences were found in the other directions. The magnitude of the horizontal CoMgl resultant lines varied according to target direction in the healthy group, while no such variation was observed in the LAS group.

### Contribution of the lower limbs and trunk to global body strategy

The contribution of the trunk and pelvis to CoMgl lowering on the SEBT task varied according to reaching direction (Figure 3A). The trunk contributed less CoMgl lowering than the lower limbs (as estimated by the pelvis lowering) in the AM direction but the contrary was seen in the PM direction. Pelvis and trunk lowering both significantly predicted CoMgl lowering for all directions and limbs. The β, represented by bars in Figure 3, was higher than 0.38 in all conditions (Figure 3B). For example, a higher β was found for the pelvis (0.82) compared to the trunk (0.38) in the AM direction (combination of the dominant and injured limbs). The opposite finding was observed in the PM direction (β: Pelvis, 0.47 and Trunk, 0.60). In contrast to trunk and pelvis, the magnitude of hip abduction of the reaching limb did not seem to contribute significantly to CoMgl lowering (Figure 3B, white bar). Finally, both segmental components seemed to have a similar contribution to CoMgl lowering in the M reaching direction.

### Combination of global body strategy variables that best predict the SEBT performance

The statistical models that best estimated SEBT performance were composed of a unique variable or a combination of a maximum of two variables (*p* < 0.05). In general, performance on the SEBT was best estimated by the magnitude of CoMgl lowering alone (8 out of the 12 conditions: 2 groups*2 limbs*3 directions; standardized Beta coefficient (β) > 0.54) or in combination with peak-to-peak vertical CoMgl velocity as the second predictor (2 conditions: the injured limb in AM direction [β for the lowering = 0.71 and the velocity = 0.52]; and the dominant limb in PM direction [β for the lowering = 0.46 and the velocity = 0.55]). Performance on the SEBT was best estimated using only the peak-to-peak vertical CoMgl velocity variable when the test was performed in AM direction with the uninjured or the dominant limb (β > 0.65) (Table 1).

**Table 1:** Associations between performances on the SEBT and global strategy variables derived from CoMgl in a single model*

Group	Reaching direction	Lower limb	Global strategy variables (global CoM in vertical plane)	Model adjusted R^2^	***p*** value	β	Partial correlation^a^
Healthy group	**AM**	Dominant	**Peak-to-peak velocity (cm/s)** ^**b**^	0.68	0.012	0.71	-
		Non dominant	**Displacement (cm)** ^**c**^	0.46	0.044	0.60	-
	**M**	Dominant	**Displacement (cm)**	0.81	0.004	0.80	-
		Non dominant		0.63	0.039	0.54	-
	**PM**	Dominant	**Displacement (cm)**	0.77	0.003	0.71	0.86
			**Peak-to-peak velocity (cm/s)**		0.014	0.52	0.78
		Non dominant	**Displacement (cm)**	0.81	0.002	0.94	-
LAS group	**AM**	Uninjured	**Peak-to-peak velocity (cm/s)**	0.68	0.019	0.66	-
		Injured	**Displacement (cm)**	0.85	0.049	0.45	0.67
			**Peak-to-peak velocity (cm/s)**		0.023	0.55	0.74
	**M**	Uninjured	**Displacement (cm)**	0.78	0.022	0.59	-
		Injured		0.45	0.023	0.72	-
	**PM**	Uninjured	**Displacement (cm)**	0.74	0.007	0.71	-
		Injured		0.54	0.009	0.81	-

*Only significant combination of variables are shown.

^a^Calculated when two strategy variables were retained.

^b^Global CoM vertical velocity (Peak-to-peak value).

^c^Global CoM lowering (Maximum value).

## Discussion

This study is the first to identify global motor strategy variables associated with performance on the SEBT. Furthermore, the results of this study demonstrate that military personnel with LAS do not use the same global strategy as healthy controls when performing a goal oriented task such as the SEBT. Their global motor strategy differed in almost all conditions (except for the uninjured limb in the medial direction), suggesting that the strategy is not only linked to the conditions where the injured limb is in stance, but also when the uninjured limb is used. This difference in global strategy is characterized by less lowering of the CoMgl and less variation in its vertical velocity. This finding highlights the importance of having a sound global motor strategy in order to perform well during the test and it also suggests that every effort should be made during rehabilitation to help recover such a strategy.

### Global motor strategy differences

According to our results, the global strategy variables along the vertical axis seem to be very good indicators of a subject’s performance on the SEBT. The chosen global strategy variables, derived from body CoM displacement, helped to characterize performance on the SEBT in terms of motor control abilities. Our findings also support the construct validity of the SEBT that maximal reach distance, as commonly measured in clinical setting, provides a valid estimate of global motor control in the studied population. The body CoM behaviour improved knowledge of how participants were organizing their movements during the critical period of stability at foot contact (central phase), allowing the transition between the two subtasks.

As supported by a systematic review and original works, performance on different motor tasks is often altered bilaterally after LAS [9–13] even in acute LAS stage [14, 15]. These differences in performance between groups could indicate a change in motor strategies as observed in the current study. The strategy used by the healthy group, leading to better performance, can be supported by biomechanical advantages and constraints. First, a lower vertical position of the CoMgl allows a better control of CoMgl horizontal displacement by increasing overall body stability. Indeed, a smaller lever arm in an inverted pendulum decreases muscle force required to maintain balance. Moreover, a lower body CoMgl position in unipedal stance increases the projection of the reaching limb by a better orientation of the pelvis, and it also increases the effect of the trunk CoM to counteract the perturbation. Thus, by a greater lowering of the CoMgl, the physical performance is optimized but the motor control requirement is much higher as the person needs to simultaneously control more body segments.

Secondly, the group with LAS demonstrated a more cautious approach near foot contact compared to their peers without LAS, as shown by a smaller peak-to-peak velocity of the body CoM. This finding suggests that LAS subjects are planning the transition between the subtasks more cautiously as they try to reduce body velocity around this event. After foot contact, a lower velocity of straightening up may allow more time to adjust for the internal perturbation and therefore enhance stability. Several studies have shown that a quick displacement of a body segment away from the body can efficiently counteract a loss of CoM control [68, 69]. In the present study, the delay in straightening up after foot contact has been attributed to the maintenance of the lower limb away from the body. Finally, global strategies in the horizontal plane also seemed to differ between groups. These variables can show changes in motor control because they give an idea of how far the participant pushed CoMgl to the perceived limits of stability, which seemed to be further in the healthy group. Moreover, the horizontal excursion of CoMgl seemed to have different characteristics according to reaching directions in the healthy group compared to the LAS group. Indeed, the LAS group showed a more grouped horizontal excursion of CoMgl for different reaching directions. This may be the result of less adaptability of the central nervous system for the group with alterations of motor control (LAS group) as previously observed for static postural control [70].

### Segmental motor strategy differences

The difference in global strategy of the military personnel with LAS was mainly explained by changes in segmental motor strategy such as a decrease in pelvis lowering during the task and a more cautious bringing back of the reaching limb. First, the LAS group, which showed the lowest performance at SEBT, seemed to lower the pelvis less by flexing the knee less, and sometimes also the ankle, compared to the healthy group. Other studies have also reported a decreased range of motion at the knee during poor performance at SEBT [45, 49, 71]. In the LAS group a significant decrease of maximal ankle dorsiflexion was found near foot contact for the AM direction only. Interestingly, it is indeed in the AM direction that the largest amplitude of ankle dorsiflexion is required [72].

Secondly, the lower peak-to-peak vertical velocity of CoMgl for the LAS group seemed to be mostly explained by a more constant velocity of the reaching limb after the foot contact and a delayed straightening up. Indeed, this smaller variation of vertical velocity in the LAS group could be partially explained by a cautious return of the reaching limb, which may help to prolong the stability advantages related to a smaller lever arm in the inverted pendulum. During the *return-from-MRD* subtask, the return to baseline velocity for hip abduction was delayed for all conditions of the injured limb of the LAS group. A more constant velocity when bringing back the reaching limb towards the body allows time to adjust the movement to preserve stability, diminishes the magnitude of the internal perturbation after a critical stability period, and also allows the use of the reaching limb to distribute mass away from the pivot point to reduce acceleration of CoMgl. Moreover, a more constant velocity of the reaching limb after foot contact is less demanding in terms of motor control compared to the bell-curve velocity profile shown in the healthy group. Indeed, a bell-curve velocity around foot contact requires excellent muscle coordination and good movement planning.

### Complementary information from the tested reaching directions

The present study confirms that each tested direction brings unique information with respect to segmental motor strategies. The non-redundancy was shown by the adaptability and differences in segmental motor strategies as a function of reaching direction along the vertical axis and in the horizontal plane. For example, the AM direction lowering, contrary to the PM direction, is better estimated by the pelvis lowering than the relative trunk lowering. This can be explained by the fact that the trunk can counteract the reaching limb less in the anterior direction by a smaller available range of motion at the hip and the back and by less powerful muscles controlling the movement in AM direction than in PM reaching against gravity [73]. In fact, a recent study has shown differences in muscle activation patterns on the SEBT according to reaching directions [47].

### Clinical implications

From a clinical point of view, the performance during the SEBT has a significant meaning, as it reflects the motor control abilities during single leg stance, the sub-phase of walking and running where LAS or giving way usually occurs. Previous studies have shown bilateral decrease in reaching distance when executing the SEBT and bilateral modifications in the characteristics of CoP displacements, thereby supporting the hypothesis of central nervous system reorganization [10, 12, 14]. However, the maximal reach distance and the measures of CoP displacement during single stance, the latter not being readily accessible to the clinician, do not provide any information on the nature of the dynamic control impairments. In the present study, we used kinematic data to describe the alterations in global and segmental strategies of persons with a LAS. Our findings not only give further support to the construct validity of the SEBT as a clinical measure of motor control, but they also indicate how alterations in motor control take place. They also provide visual cues that give insight on how movement should be retrained. As reaching with one limb while standing on the opposite foot may also be used as a challenging rehabilitation exercise, it might be wise for a clinician to ask the patient to reach for an object located on the lateral side of the stance foot to force a lateral displacement of the CoMgl towards the limits of the base of support. One could also stimulate the lowering of the CoMgl by asking the patient to further bend the lower limb while reaching as far as possible with the free limb.

### Study limitations

In the present study, several variables were measured using a fairly small sample size of participants, and this may have limited the possibility of showing significant differences in some global strategy variables, especially the ones in the horizontal plane where a high inter-participant variability and differences in foot length could have had an impact. Also, while we ensured that both groups had similar personal characteristics, participants in the two groups were not individually paired. Therefore, it was not possible to control for side of injury and limb dominance in our comparative analyses. As previously mentioned, considering the high proportion of LAS injuries to the dominant limb, it was decided to use the dominant limb of the participants in the healthy group for intergroup limb comparisons. By doing so, the differences in the uninjured limb of the LAS group may have been slightly overestimated. Finally, it is important to mention that the calculated global body CoM is an estimate of true body CoM. Anthropometric segmental components of the body of each participant were not incorporated into the calculation of body CoM but were taken from population averages. This could have led to a slight over- or under-estimation of CoM position, even if theoretically it should have affected both groups equally.

## Conclusion

Compared to healthy subjects, individuals with LAS had a poorer performance and used a global strategy on the SEBT involving different segmental strategies: less vertical displacement mainly by less pelvis lowering and lower range of motion in knee flexion than the individuals without LAS. A smaller peak-to-peak of vertical velocities of CoMgl for the LAS group seemed to be mostly supported by a delayed straightening up and a more constant velocity return of the reaching limb after foot contact. The present study is the first to document bilateral differences in global motor strategy as well as in performance on the SEBT after a very common and highly prevalent musculoskeletal injury. The present findings suggest that rehabilitation after a local injury, namely a LAS, should address global task training and use the specific variables that are more likely to accelerate and enhance motor control recovery.

## Endnote

^a^Winter equation: (2*0.0145*foot*) + (2*0.0465*calf*) + (2*0.1 *thigh*) + 0.497*trunk* + 0.081*head* + (2*0.028*upper arm*) + (2*0.022*forearm*) = 1.0

## Authors’ original submitted files for images

Below are the links to the authors’ original submitted files for images. Authors’ original file for figure 1 Authors’ original file for figure 2 Authors’ original file for figure 3 Authors’ original file for figure 4 Authors’ original file for figure 5
