# Supplementary figures and images for: Alteration in global motor strategy following lateral ankle sprain
Source: BMC Musculoskelet Disord. 2014 Dec 16;15:436. doi: 10.1186/1471-2474-15-436 (PMC4300726; doi:10.1186/1471-2474-15-436)

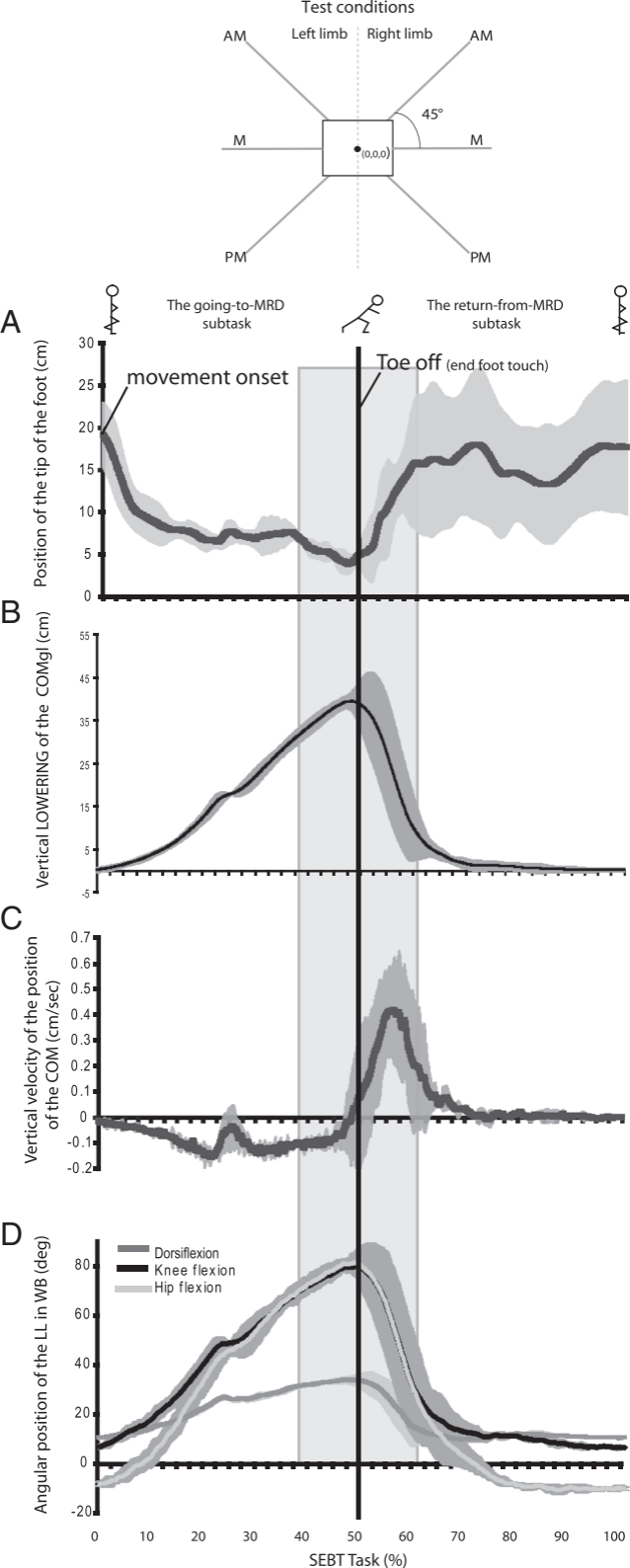

Supplement: Supplementary file 1 — Authors’ original file for figure 1 [file 12891_2013_2367_MOESM1_ESM.pdf]

**A**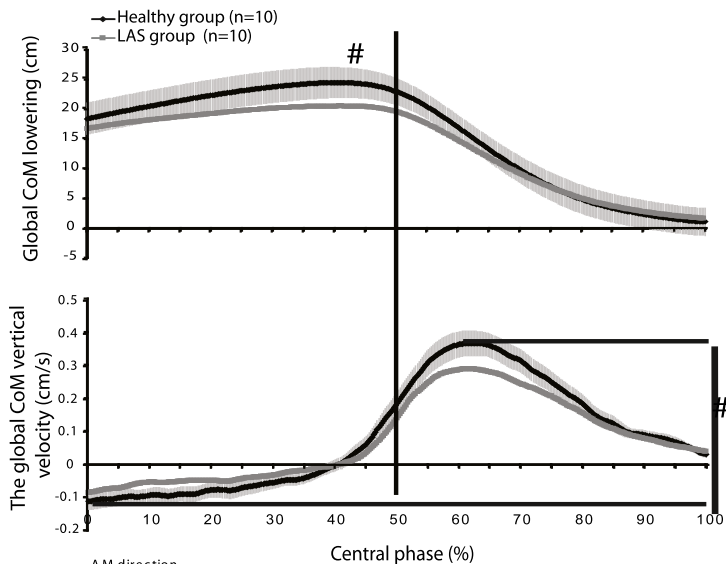**B**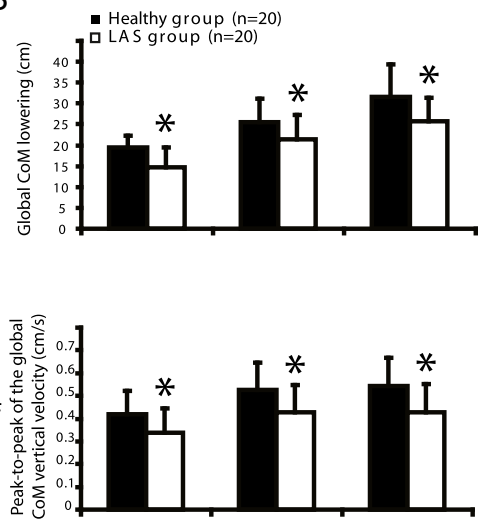**C**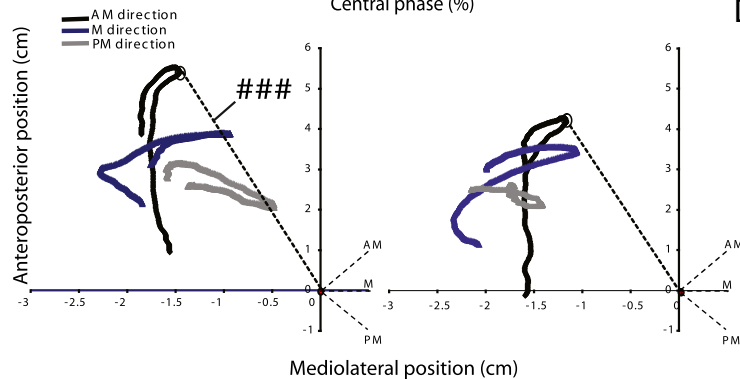**D**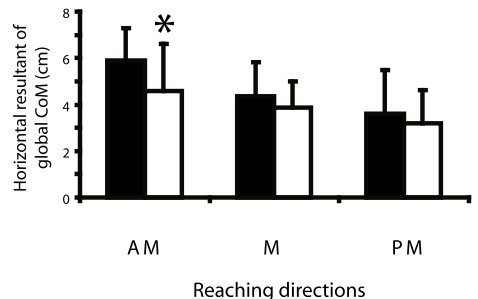

Supplement: Supplementary file 2 — Authors’ original file for figure 2 [file 12891_2013_2367_MOESM2_ESM.pdf]

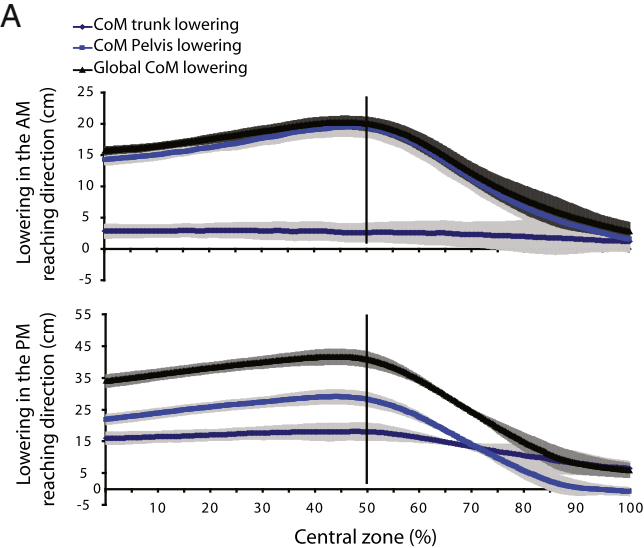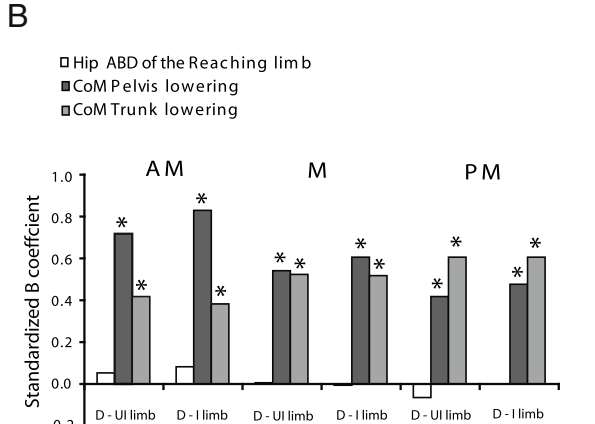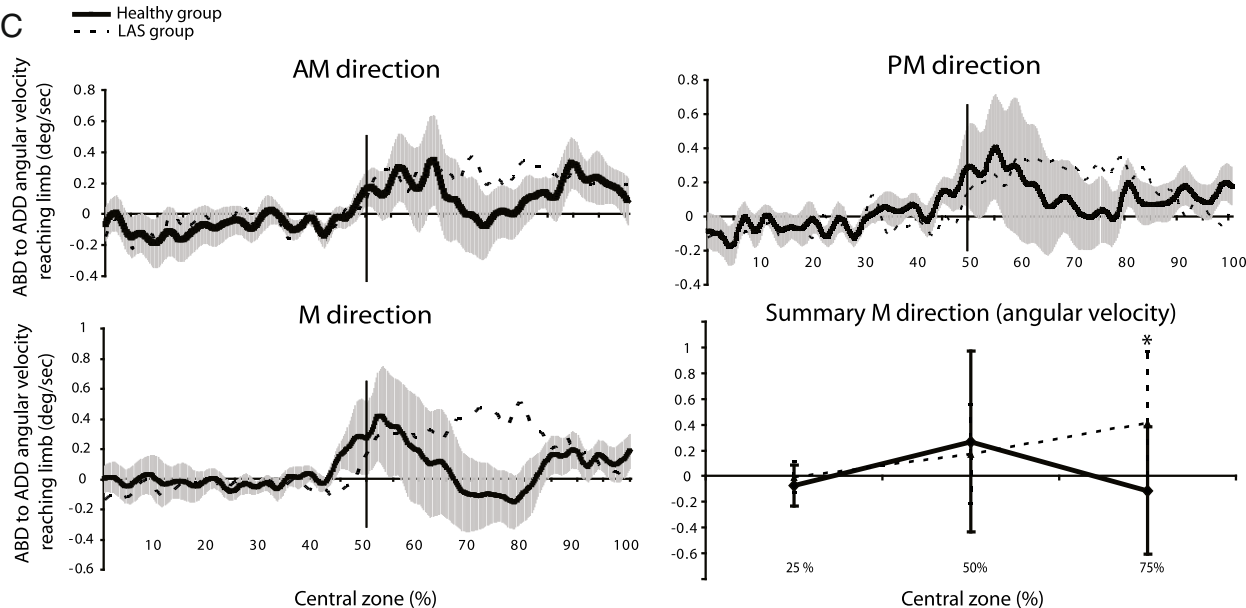

Supplement: Supplementary file 3 — Authors’ original file for figure 3 [file 12891_2013_2367_MOESM3_ESM.pdf]

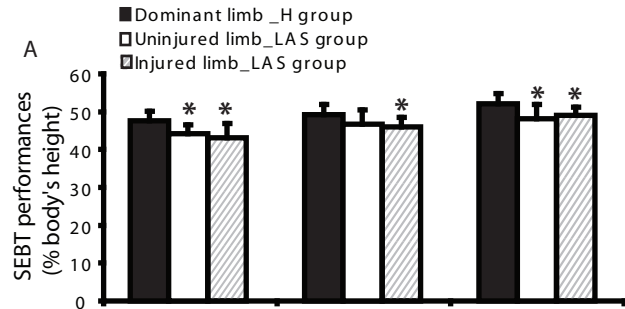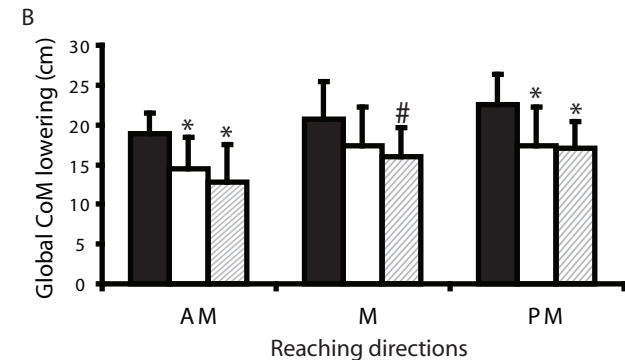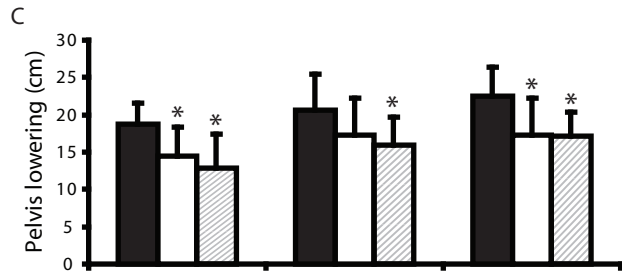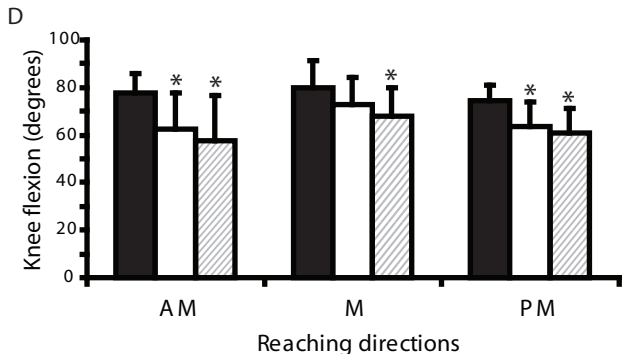

Supplement: Supplementary file 4 — Authors’ original file for figure 4 [file 12891_2013_2367_MOESM4_ESM.pdf]

**Figure 4:**


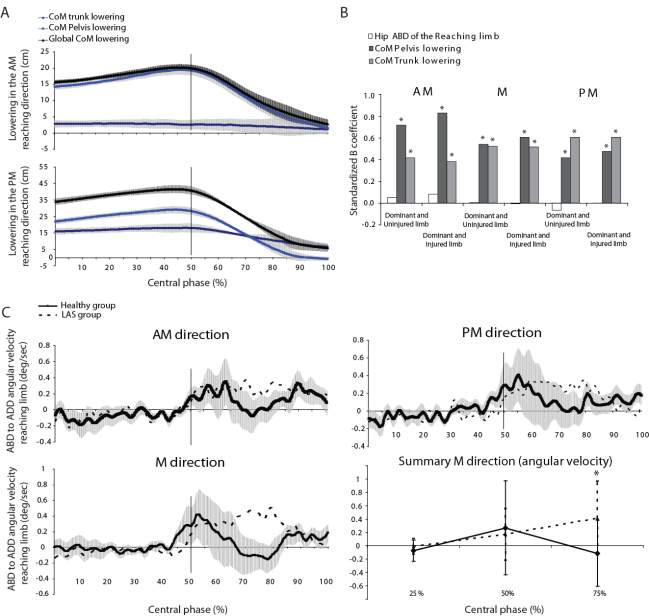

Supplement: Supplementary file 5 — Authors’ original file for figure 5 [file 12891_2013_2367_MOESM5_ESM.doc]
